# Rebranding of Predatory Journals and Conferences: Understanding Its Implication and Prevention Strategy

**DOI:** 10.7759/cureus.40126

**Published:** 2023-06-08

**Authors:** Alessandro Martinino, Surobhi Chatterjee, Frank W Smeenk, Sjaak Pouwels

**Affiliations:** 1 Surgery, University of Illinois at Chicago, Chicago, USA; 2 Internal Medicine, King George's Medical University, Lucknow, IND; 3 Health Professions Education, Maastricht University, Maastricht, NLD; 4 Intensive Care Medicine, Elisabeth-Tweesteden Hospital, Tilburg, NLD

**Keywords:** scientific publishing, predatory journals, fake conference, ethics, bootlegging, rebranding

## Abstract

Academic conference participation and publications serve as a litmus test to evaluate researchers irrespective of their scientific discipline. Predatory or fake conferences and journals exploit this issue and rebrand themselves through multiple methods. This paper serves to introduce rebranding as one of the features adopted by predatory journals and conferences and formulate some important measures that could be taken by academic libraries, researchers, and publishers to address this issue. We found that rebranding serves as an efficient measure to evade legal implications. However, empirical longitudinal studies addressing the issue are absent. We have explained rebranding, multiple ways of rebranding, issues surrounding predatory publishing, and the role of academic libraries and provided a five-point prevention strategy to protect researchers from academic malpractices. Dedicated tools, scientific prowess, and vigilance of academic libraries and researchers can safeguard the scientific community. Creating awareness, increasing transparency of available databases, and the support of academic libraries and publishing houses along with global support will serve as effective measures to tackle predatory malpractices.

## Introduction and background

Predatory publishing has disrupted the scientific integrity and robustness of scientific research. Though there is no comprehensive definition of predatory publishing at present, in the words of Jeffrey Beall, predatory journals are the ones "that exploit the author-pays model, damage scholarly publishing and promote unethical behaviour by scientists" [1]. He first curated the famous Beall's list in his blog Scholarly Open Access in 2010. Predatory conferences are another add-on to the list wherein usually early career researchers are invited through personal emails and provide opportunities to publish their work as conference proceedings [2] after publicizing eminent experts as keynote speakers without their knowledge [1-3]. The critique of predatory publishing lies in advancing economic pursuit and not science. The line seems to be blurred with some traditional in-house low-funded publishing companies not performing robust peer reviews after charging article processing charges upfront [4] or relying on, most of the time, free inexpert advice. There are multiple focus points regarding predatory academic publishing but one such merits discussion. In the recently published editorial by Siler et al., a dangerous cybercrime was presented in detail, namely, bootlegging and rebranding of papers [5].

While this recent work focused on rebranded predatory journals, we would also like to address a step further to understand "what is rebranding" and the "why" behind it and provide suggestions on "what can be done about this problem" and the "role academic libraries and other stakeholders can play" for predatory publishing, predatory conferences, and rebranding.

## Review

Multiple methods of rebranding

Rebranding serves as an efficient system of hijacking both the scientific and judicial systems of a country. Hijacked conferences are less addressed than hijacked journals [3,6]. There are several ways in which publishing groups can manipulate early career researchers. The first way is by creating “sister pirate websites” or "hijacked websites" through which low-budgeted conferences in developing nations and even branded conferences can be easily replicated [3]. This has led to huge issues wherein search results are manipulated and boosted, creating dilemmas and confusion between fake and real conferences [3]. Thus, many established conferences have now started to provide a disclaimer claiming authenticity like the American Association of Neurology and European Academy of Neurological Sciences among others. The second way is through “rebranding,” wherein some of the established, but poorly funded publishing groups, are bought by predatory publishing groups, for instance, the takeover of the Pulsus Publishing Group by OMICS [7]. Now, this takeover is neither represented in the official site for Pulsus conferences (publicizes itself as a legal and popular entity, utilizing its previous track record) which continues to operate despite legal brandishing of OMICS group nor on the OMICS website [7]. Despite being sued by the US Federal Trade Commission in 2019 for making false claims and hiding publishing fees, OMICS continues to hold conferences in multiple regions of the world through the several networks it has created, namely Conference Series, Pulsus Group, EuroSciCon, and Life Science Events [7]. Another way of rebranding is through bootlegging or copy-pasting papers published in one established journal to a predatory journal verbatim. Siler et al. reported, "at least nine papers in the Journal of Bone Research and Reports, published by an iMEDPub LTD, had been lifted directly from the Elsevier journal Bone Reports" [5]. The hijacking of branded journals by creating counterfeit and replicable sites is another addition to the list of cybercrimes [8].

Detriments of predatory journals and conferences

The lackadaisical attitude of the judicial system in the host country provides a safe haven for predatory publishing networks [9]. The dangers of these papers are not just restricted to publishing houses and authors who are virtually reprinting the materials and serving them but also to the patients and caregivers themselves [9]. When freely served, manipulated pseudoscience gets propagated and consumed, and it can significantly harm the well-being of patients [9]. Another reason for the inaction is the myth that only early career researchers are doomed to fall into the trap [4,6] and the lack of initiatives to create awareness in them. Early scholars are also blinded by the fact that they are invited to a global conference [3]. The “publish or perish” academic environment and lack of awareness of the gimmicks adopted by these publishing houses leads to these widespread problems [3,9]. Some of the eminent scholars are even unaware that their names and affiliation are being used by these predatory conferences [1,2]. One of the strategies adopted includes personalized emails highlighting previous research papers which provide a boost to the self-esteem of early career researchers who are lost in the belief that they are being "celebrated" for their published works [2,6]. Another often overlooked reason is the lack of continuous scrutiny of acclaimed databases like PubMed [10,11] and Scopus [12] which at times also leads to dysregulation of these predatory publishers who easily rebrand and get their journals included by taking advantage of the loopholes. Some studies have reported that even PubMed and Scopus index several predatory journals [10-12]. Such highly accredited and reputable indexing is an efficient measure to persuade the researcher.

The hot spots for predatory publishing

India is considered the “hotbed” of predatory publishing and conferences [6]. Both an academically developing system, lack of awareness among early researchers, use of publication metrics for promotions and tenures, and limited funding made India the global hotspot [4]. A longitudinal analysis after sampling 613 journals out of 11,000 identified predatory journals indicated that out of 262 sampled authors who published in predatory journals, the majority were from developing countries including India (34.7%), Asia without India (25.6%), and Africa (16.4%) [13]. However, reports have suggested that not just low-middle-income countries (LMICs), but even researchers from affluent countries as well frequently publish in predatory journals [14].

Role of academic libraries

The Current State of Understanding and Challenges

Academic libraries are the pillars of scholarly communication whose three caveats are evaluation, dissemination, and preservation [15]. Predatory publishing undermines all three. There is limited to no peer review in the publications, but their dissemination is easier because of easier accessibility options than standard publishers by exploitation of the Creative Commons licensing [16]. They even create fabricated journal metrics like impact factor and indexing [9]. The preservation strategies, including archival status and long-term availability, are not safeguarded [16]. Rebranding adds to the already present dilemma in classification as most of them mention DOI, CrossRef, and Data cite [9,16,17] and then rebrand the published papers keeping no association with the former journal in which the paper was originally published [5].

Existing Challenges for Academic Libraries

At present, most libraries still rely on the now-defunct “Beall's list,” which has drawn criticism due to a lack of transparency, robustness, and limited updates [18]. The subscription access tool “Cabell's whitelist and blacklist” is well curated, has more ethical standards, and is updated but has prohibitive costs for institutional and individual access [19,20]. Cabell's whitelist has limited verifiability of its inclusion criteria and limited indicators related to the transparency of the journals [20]. The “Directory of Open Access Journals (DOAJ)” [21] is a community-curated list of open-access journals but is not a complete inventory of all legitimate open-access journals, and it uses limited criteria while quantifying peer review and editorial services [20]. The “World Association of Medical Editors (WAME)” created a “predatory journal algorithm” based on Beall's list and DOAJ, with each having significant limitations of its own [22]. In addition, there are several overlaps between the whitelists (Cabell and DOAJ) and the blacklists (Beall and Cabell) which point out that many journals are misclassified or fall in the grey area [20]. There is a huge gap between the availability and understanding of rebranding and predatory publishing in the present academic librarianship environment versus the pace at which predatory publishing is rapidly flourishing and taking advantage of the loopholes [22].

Novel Initiatives Taken Up by Academic Libraries Globally

To address the current lacunae, a commendable innovation in the form of the “Scholarly Tools Opposing Predatory Practices (STOPP)” tool is developed by the University of Texas librarians. STOPP is a unique tool, which addresses not only publisher websites but also email solicitations, conferences, and thesis converters [16]. There are four tools that are part of the STOPP tool, each requiring answers to [20] dichotomous questions (Yes or No) for classification, namely “Conference Assessment tool,” “Email assessment tool,” “Thesis Convertor assessment tool,” and “Website assessment tool.” The training of academic librarians on this tool provides them the confidence to better understand and address predatory publishing [16].

Future Directions for Academic Libraries

Academic librarians need to be well-trained in the challenges faced in academic publishing. It is imperative that a robust, continuously updated tool is available for free usage to address the challenge globally. Academic librarians are often referred to determine whether a publisher or journal is predatory or not [16]. Thus, they need to understand predatory publishing, rebranding, and bootlegging to advise universities and make recommendations. There is a future need for the development of practical tools to empower librarians and researchers in reaching conclusions about whether to partner with a potential outlet for dissemination [16]. There is an immediate need to create a widely accessible, freely available tool or application to address rebranding as the majority of the predatory outlets are based in developing countries wherein high subscription costs will be prohibitive [4].

The five-point prevention strategy

Based on a thorough literature search of academic papers on the subject [3,4,6,9,13,16,20,23] and after a careful analysis of predatory conferences and journal invites we receive as early career researchers, we have come up with a few suggestions to tackle this issue. Our suggestions are based on five important prerequisites: an accessible rigorously updated tool or freely available checklist for deciding upon predatory publishing, second on increasing the robustness of the available indexing, third on the role of academic libraries in creating awareness on the issue, fourth on the need for academic publishing houses to be more inclusive, and fifth on global support.

Checklist and Tools for the Identification of Predatory Publishing and Conferences

In the short term, editorial boards and data scientists need to collaborate and provide a freely accessible and robust checklist to ascertain predatory publishing by scholars, academic librarians, and universities (like EQUATOR guidelines available to guide the various study types). Shamsher et al.'s list serves as an essential resource [24]. Beginning with promoting the available Think.Check.Submit [25] and Think.Check.Attend tools [26] for predatory journals and conferences, respectively, is imperative. A web-based and application-based tool could be developed and disseminated for free access to ascertain the potential of being a predatory journal. There is a need to address predatory conferences as well where few developments have been made [3]. The tool needs to be updated frequently, and it can protect both scholars and readers from such academic malpractices. In the long term, there is a need for the conglomeration of the various databases available on predatory publishing and conferences like the available blacklists and whitelists [19] and including work done at smaller levels like through OSF Collaboration or Kscien after effective decisions on the definition and understanding are made [26,27]. This could be an open-access crowd collaborator interface much like the Cochrane Crowd Collaboration of reviewers albeit for predatory publishing and conferences wherein all the researchers could simply update the details of the emails/invitations they have received from predatory journals and conferences and fill in some basic details. They could then be regularly updated and reviewed by the lead group if publishing groups and/or researchers complain about the veracity of the inclusion. These prepositions, if added to the current work being done, could have a significant impact in hampering the way predatory publishing and re-branding function.

Increasing Robustness of Available Indexing

For improving existing indexing policies of PubMed, MEDLINE, SCOPUS, EMBASE, EBSCO, and Thomson Reuters (now Clarivate Analytics) etcetera, quality control should be reinforced during the application process [28,29]. The whole process available for inclusion should be clear and understandable for the editorial boards of the journals. The reviewing process needs to include a thorough background check of the journal including the editorial board, previous publications, and peer review policies [11,28]. For instance, after reporting a journal, even though SCOPUS removes the journal, the article added to their repositories till the date of delisting remains visible, inflating the author-level metrics [29]. Alongside, regular scrutiny and an effective complaint system to address predatory journals and the removal of the articles published in them to date [29] will ensure that the available reliable databases like PubMed, EMBASE, Scopus, and Web of Science among others are free from academic malpractices.

At present, the discriminatory attitudes lead to the inclusion of predatory journals supported by publishing houses in PubMed [11] and Scopus [28,29] who know the loopholes of indexing but not student-led and supported journals [30], which leads to their discontinuation after a period of time due to lack of incentives and academic and industrial support. The limited to no student journals indexed in PubMed despite years of ethical publication are clear examples [30]. In addition, numerous hybrid specialties crossing with the world of healthcare, such as engineering, management, and economics, may encounter the same scenario. In fact, in these disciplines, many longstanding and recognized journals may not be indexed. In this context then, where indexing cannot be completely relied upon, the potential tools discussed in the section "Checklist and Tools for the Identification of Predatory Publishing and Conferences" gain even more value and can be positively identified as concrete solutions to differentiate the morality and ethicality of the journal considered, elevating it from the predatory ones.

Academic Libraries: The Multitude of Roles They Can Play

From ensuring information dissemination and awareness to safeguarding patrons, academic libraries can play multiple roles. In the immediate term, academic librarians need to keep themselves well updated on the different journals and conferences [16] and maintain a regional database of the reported or identified predatory groups. In the short term, the creation of innovative tools like STOPP could help ease the process of identification of predatory conferences, websites, journals, and even emails [16]. However, in the long term, the dissemination of knowledge and safeguarding services and legal framework need to be created with community efforts [16].

Academic Publishing Houses and Their Role

The birth of predatory publishing relied heavily on the inception of the Open Access (OA) publishing strategy [1,5,9]. Though most reputable journals now are either only OA or have an option of being subscription-based or OA, the modus operandi of these predatory publishers relies heavily on the exploitation of OA publication strategy for economic pursuit and not on the dissemination of science [9]. Gold OA and faster publication along with our reliance on them for academic promotions push researchers to get published [9,29]. Hence, they knowingly or unknowingly fall prey to predatory publishing [4,9].

Global Cooperation and Support

An immediate short-term action can be through the network of medical schools/universities in these "hot" countries and by providing fee waivers or APC discounts to researchers from LMICs [29]. We should encourage global organizations or networks to create and promote free materials to educate the new generations in these universities. Finally, this is a global call for the entire strata of the scientific community, including researchers, authentic publishing groups, and government organizations to make common grounds, penalties, and universal laws governing scientific publication [9]. There needs to be a proper legal framework and charges on publishing groups so that the current pace of rebranding is halted [5,9].

## Conclusions

Predatory journals and conferences represent an obvious problem, which needs to be addressed both in the short and long term by the scientific community. Open Access and faster publication, along with the need of being productive as researchers in the academic environment, represent some of the main reasons for their rapid spread. An accessible rigorously updated tool for deciding upon predatory publishing, increasing the robustness of the available indexing, and dissuading and creating awareness on the issue globally could represent different solutions to address the problem.
